# Safety and Efficacy of a “High and Low Molecular Weight Hyaluronic Acid Hybrid Complex” Injection for Face Rejuvenation

**DOI:** 10.1111/jocd.70117

**Published:** 2025-04-09

**Authors:** Taraneh Yazdanparast, Azin Ayatollahi, Aniseh Samadi, Araz Sabzvari, Hamidreza Kafi, Alireza Firooz

**Affiliations:** ^1^ Center for Research & Training in Skin Diseases & Leprosy Tehran University of Medical Sciences Tehran Iran; ^2^ Medical Department Orchid Pharmed Company Tehran Iran

**Keywords:** dermis density, dermis thickness, elasticity, high and low molecular weight, hyaluronic acid, hydration, TEWL

## Abstract

**Introduction:**

Hyaluronic acid (HA)‐based formulations could have remarkable efficacy in treating a wide range of skin defects, including skin aging. The purpose of the study was the evaluation of the clinical safety and efficacy of a high and low molecular weight HA hybrid complex injection for skin rejuvenation.

**Method:**

In this single‐arm, before‐and‐after clinical study, 20 subjects with wrinkled, dry, or rough skin were enrolled. They received two treatment sessions, each of 2 mL of stabilized high‐ and low‐molecular‐weight HA through intradermal injections in five bioaesthetic points with an interval of 4 weeks. Efficacy assessment measures included biophysical and sonographic parameters and the Global Aesthetic Improvement Scale (GAIS) score. Pain assessment, safety profile, and subject satisfaction were also reported.

**Results:**

A significant improvement in skin firmness was demonstrated in both follow‐up visits. The Transepidermal water loss (TEWL) and the dermis ecodensity improved significantly in the first follow‐up visit. A statistically significant increase in the dermis thickness was seen in the second follow‐up visit. The median GAIS score indicated an average improvement of 51%–75%. The median overall satisfaction score was 7 and 6 in the first and second follow‐up visits. No important side effects were observed. The average pain VAS score was 2 out of 10.

**Conclusion:**

This new HA‐based formulation is a safe and efficient treatment option to restore the vitality and turgidity of the skin.

**Trial Registration:**

ClinicalTrials.gov identifier: IRCT20150101020514N17 with ethics code IR.TUMS.TIPS.REC.1401.083

## 1 Introduction

Facial rejuvenation is a rapidly progressing field in aesthetics, and minimally invasive procedures are powerful tools to achieve it [1]. The positive effect of minimally invasive panfacial treatment extends beyond improving physical appearance and improves social perception and observer‐reported outcomes [2].

Aging causes complex but predictable changes. There are different rates at which these alterations affect the face, based on extrinsic and intrinsic factors, including sun exposure, smoking, air pollution, underlying diseases, genetics, ethnicity, etc. [1]. The signs of facial aging make an individual look older, miscommunicate a mood of sadness or anger, affect social interactions and perceptions, and self‐esteem [3].

Smoothness, high elasticity, and evenly colored are the characteristics of healthy and attractive skin [4, 5]. The reduction in the synthesis of dermal extracellular matrix components and the reduction in hyaluronic acid (HA) due to decreased fibroblast activity is one of the main skin aging processes [6]. This process leads to the loss of skin elasticity and turgidity [7].

HA, a dermal polysaccharide that maintains the skin's hydration and induces the proliferation of dermal cells, has many properties that make it more appropriate for skin rejuvenation than other substances [8]. It is one of the compounds most often used to restore skin appearance and has shown a high efficacy and good safety profile in clinical studies [9]. A thorough analysis of the literature demonstrated that HA‐based formulations have shown remarkable efficacy in treating a wide range of skin defects, including wrinkles, nasolabial folds, and skin aging. This has been achieved via soft tissue augmentation, skin hydration improvement, collagen stimulation, and face rejuvenation [10].

The product studied (Perleux, Espad Pharmed, Iran) is a buffered physiological solution containing high molecular weight (H‐HA) and low molecular weight (L‐HA) HA. The purpose of this study was to investigate the clinical safety and efficacy of the product's injection in facial bioaesthetic points, for skin rejuvenation and improvement of skin biophysical parameters.

## 2 Method

### 2.1 Participants and Study Design

From January to June 2023, 20 males and females with wrinkled, dry, or rough skin with low elasticity, Fitzpatrick skin types III and IV, and ages ranging from 35 to 60 years were enrolled in this single‐arm, before‐and‐after clinical trial. The study center was the Center for Research and Training in Skin Diseases and Leprosy, Tehran University of Medical Sciences, Tehran, Iran.

The exclusion criteria were as follows: pregnant or lactating women; treatment with HA‐based dermal fillers within the last 12 months; rejuvenating laser, radio frequency, botulinum toxin injection, or intense pulsed light in the last 6 months; systemic diseases affecting skin health; using systemic medications affecting the skin, such as glucocorticoids, isotretinoin, immunomodulators, and hormonal drugs in the last 3 months; taking supplements containing collagen, HA, or vitamin C in the last 3 months; using topical corticosteroids or retinoids in the last 4 weeks; using topical cosmetic products containing anti‐aging ingredients in the last 2 weeks; active smoking during the last 2 years; major changes in lifestyle, including diet and physical activity and sun exposure during the study; having an allergy or sensitivity to HA products; active inflammation, infection, or unhealed old wounds and skin lesions in the injection area; autoimmune or immune deficiency diseases or using any immunosuppressive drugs; current treatment with anticoagulation therapy or using non‐steroidal anti‐inflammatory drugs or other substances known to increase coagulation time within 7 days before enrollment; history of anaphylactic shock; being prone to hypertrophic scar formation; and having unrealistic expectations.

### 2.2 Product

The test product constitutes a buffered physiological solution of H‐HA and L‐HA (2 mL of stabilized HA). It is a sterile and pyrogen‐free medical device for intradermal use in the form of a pre‐filled glass syringe with two needles 30G × ½, and it contains 32 mg of H‐HA and 32 mg of L‐HA in 2 mL of buffered sodium chloride physiological solution. The pre‐filled syringes are sterilized by moist heat. Needles are sterilized with ethylene oxide. The used HA is produced through the biosynthesis of a natural substrate without further chemical modification, resulting in excellent tolerability.

Moreover, because of a specific and patented treatment of the product (Hybrid Technology), the H‐HA and L‐HA chains interact with each other providing unique rheological characteristics and thus allowing the administration of higher concentrations of HA without increasing the viscosity.

The product's formulation, combining HA of different molecular weights, is based on the Hydrolift Action concept. Hydrolift Action is a complete anti‐aging concept aimed at hydrating. This new method has targeted counteracting the physiological reduction of HA in the skin, restoring elasticity, skin tone, hydration, and finally the mechanical action of lifting the skin.

### 2.3 Intervention

The intervention process consisted of two treatment sessions with an interval of 4 weeks and with the high‐ and low‐molecular‐weight hyaluronic acid hybrid complex.

**FIGURE 1:**
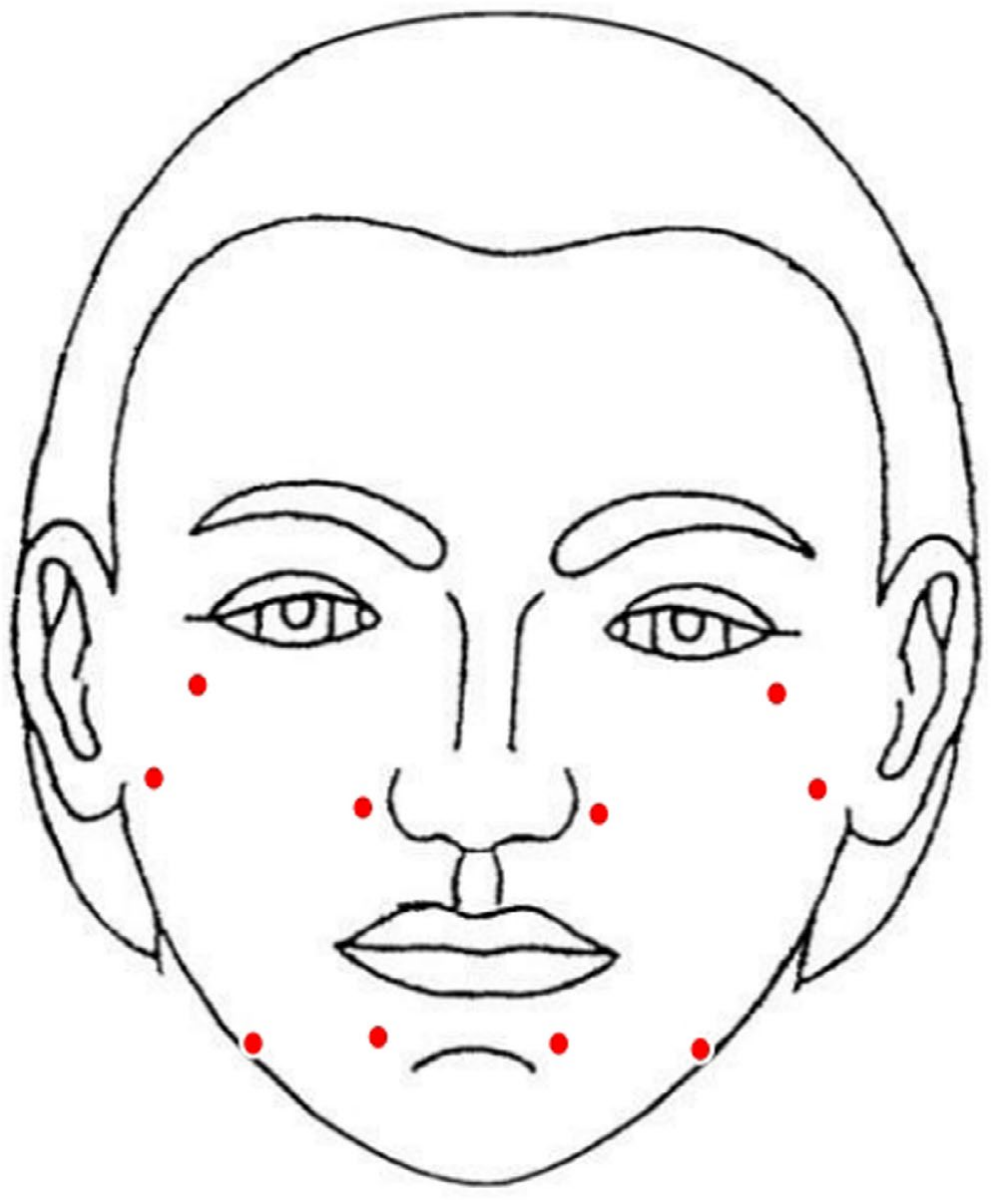
Representative schematic figure of injection points of the face (zygomatic protrusion, nasal base, tragus, chin, and mandibular angle).

After obtaining written informed consent, five bioaesthetic points—zygomatic protrusion, nasal base, tragus, chin, and mandibular angle—were selected for intradermal injection on each side of the face (Figure 1). At each point, 0.2 mL of the product was injected with the bolus technique in the deep dermis.

Anesthetic lidocaine 2%–prilocaine 2% cream was used in the form of an occlusive dressing 30 min before injection to reduce pain. The treatment site was completely disinfected with povidone‐iodine before the injection. After the injection, the treated area was massaged by the dermatologist for better distribution of the product. Then, an ice compress was applied for a few minutes. The subjects stayed at the study site for half an hour so that if there was a reaction to the injection, the research team could take the necessary actions. The post‐injection recommendations were given to the participants in writing.

### 2.4 Assessments

The primary outcome was the efficacy evaluation of the product on skin elasticity of the right cheek (on the zygomatic bone protrusion) at 4 and 12 weeks after the second injection. Skin elasticity was evaluated with a Cutometer MPA 580 (C + K Electronics), an instrument to assess the skin's elastic properties using suction and elongation. The investigated parameters included R0 (total elastic and plastic deformation or firmness), R2 (gross elasticity), and R5 (net elasticity) [11].

The secondary outcomes evaluated at Weeks 4 and 12 after the second injection included the general condition of the face in terms of signs of skin aging, with the standard before‐and‐after photographs evaluated by two independent physicians and using the Global Aesthetic Improvement Scale (GAIS) score; thickness and density of the dermis measured by a 22 MHz ultrasound probe of the DUB skin scanner device (tpm Company, Luneburg, Germany); stratum corneum (S.C) hydration; transepidermal water loss (TEWL); and friction by the relevant probes of the MPA 580 device measured on the right cheek; subject satisfaction; procedural pain assessment; and safety profile.

GAIS score is a 5‐point Likert scale as follows: 0 = No improvement, 1 = ≤ 25% improvement, 2 = 26%–50% improvement, 3 = 51%–75% improvement, and 4 = 76%–100% improvement [12]. Pain intensity during injection was measured using Visual Analogue Scale (VAS) from 0 to 10. Consequently 0 indicated complete painlessness and 10 indicated maximum pain imaginable. Overall satisfaction was evaluated using a 10 point VAS and to determine subjects' assessment of the improvement in their skin conditions (including hydration, firmness, brightness, and softness), the validated questionnaire of Sparavigna et al.'s article was used [13].

### 2.5 Statistical Analysis

SPSS 24 statistical software was used for data analysis, and *p* value < 0.05 was considered statistically significant in all analyses. Data were expressed as mean and standard deviation (SD), median and range, and percentages as appropriate. The efficacy of treatment was assessed by comparing the mean/median values of each parameter in the sessions before and after the intervention using the paired sample *t*‐test or the Wilcoxon test.

## 3 Results

Twenty subjects were enrolled in the study, including 18 (90%) women and 2 men (10%) with the age range of 37–57 years (mean ± standard deviation: 46.90 ± 5.77 years). One of the participants did not return for the last follow‐up visit. The total amount of the injected product was 2 mL in either of the two treatment sessions.

As shown in Table 1, the changes in the R0 parameter (skin firmness) showed a significant improvement at Weeks 4 and 12 after the last treatment (*p* value < 0.01). No significant change was observed in the R2 and R5 parameters in either of the two follow‐up visits.

**TABLE 1:** Skin biophysical and ultrasonographic measurements, before intervention, 4 and 12 weeks after the second treatment.

Variable (Unit)	Baseline	Week 4 after second treatment	Week 12 after second treatment	*p* *	*p* **
	Mean ± SD				
R0 (firmness) (mm)	0.25 ± 0.05	0.17 ± 0.04	0.14 ± 0.03	**< 0.01**	**< 0.01**
R2 (gross elasticity) (%)	0.63 ± 0.0	0.60 ± 0.12	0.65 ± 0.13	0.153	0.582
R5 (net elasticity) (%)	0.21 ± 0.08	0.19 ± 0.06	0.23 ± 0.11	0.482	0.477
TEWL (g/m^2^/h)	13.37 ± 2.67	11.56 ± 2.31	16.62 ± 9.15	**< 0.01**	0.129
Hydration (arbitrary)	34.83 ± 10.71	38.58 ± 6.75	40.91 ± 17.21	0.182	0.153
Friction (arbitrary)	608.44 ± 200.29	553.17 ± 192.17	559.36 ± 291.11	0.332	0.602
Thickness of dermis (μm)	1140.38 ± 340.94	1279.30 ± 326.08	1429.68 ± 227.48	0.249	**< 0.01**
Density of dermis (arbitrary)	34.58 ± 11.75	41.15 ± 14.89	37.02 ± 11.26	**< 0.01**	0.244

*Note:* Bold values are statistically significant.

* Compares the **baseline** results with Week 4 and the *p* value.

** Compares the **baseline** results with Week 12 after the second treatment (paired sample *t*‐test).

Changes in other biophysical parameters, including the S.C hydration, friction, and TEWL on the right cheek area, are shown in Table 1. Except for the significant improvement of TEWL at Week 4 after the last treatment (*p* value < 0.01), no significant changes were observed in S.C hydration and friction.

**FIGURE 2:**
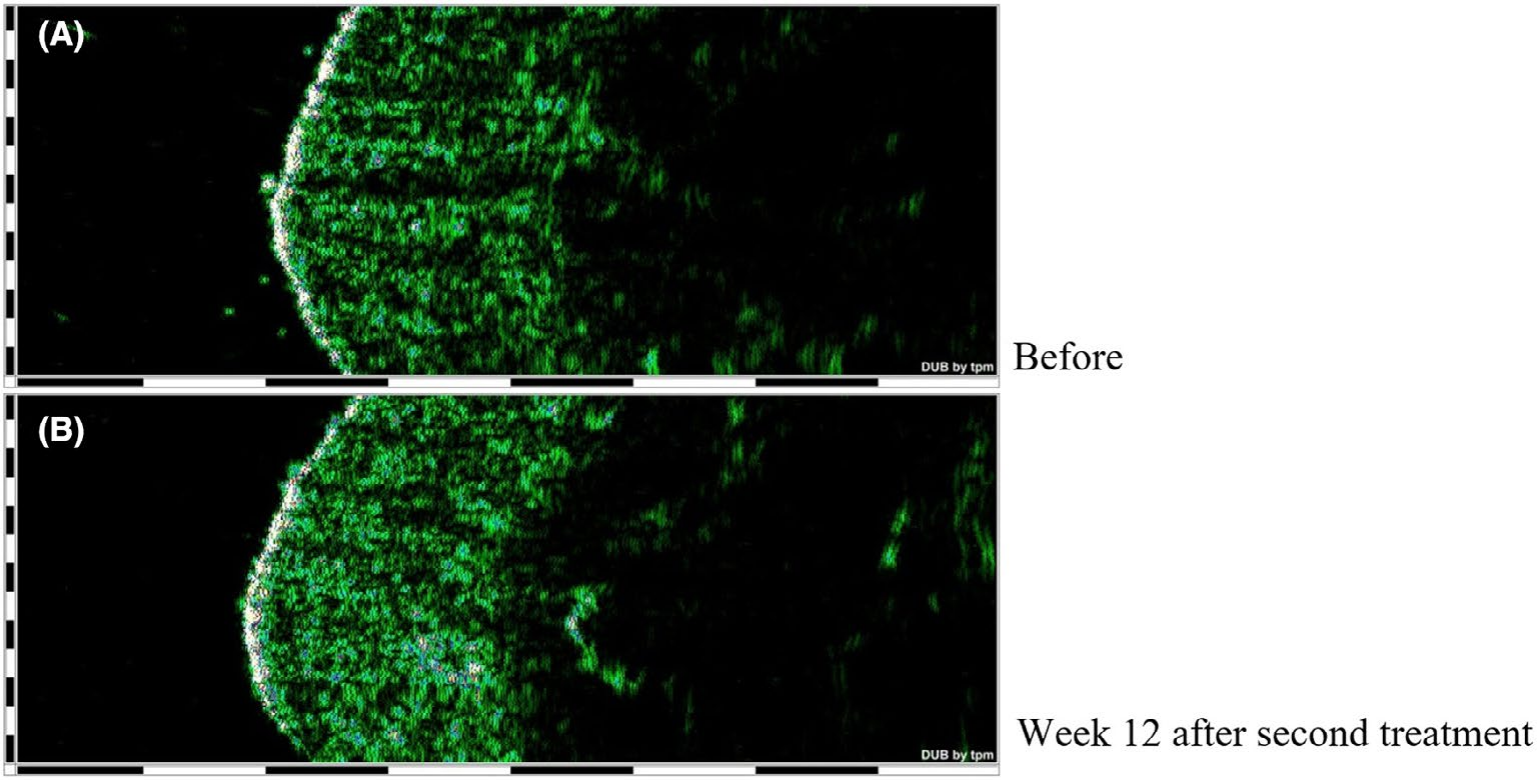
Dermis thickness (unit: μm) and density (unit: Arbitrary) improvement in a 44‐year‐old man, evaluated by 22 MHz ultrasound probe (A: Before treatment/B: Week 12 after second treatment).

Table 1 also shows the comparison of dermis thickness and density before and after the treatment. An increase in the dermis thickness was seen in both follow‐up visits, and this increase was statistically significant at Week 12 after the second injection (*p* value < 0.01). The ecodensity of the dermis layer showed a significant increase at 4th week (*p* value < 0.01) and a non‐significant increase at 12th week (*p* value: 0.244) after the second injection. Figure 2 shows the improvement of dermis thickness and density in one of the cases.

**FIGURE 3:**
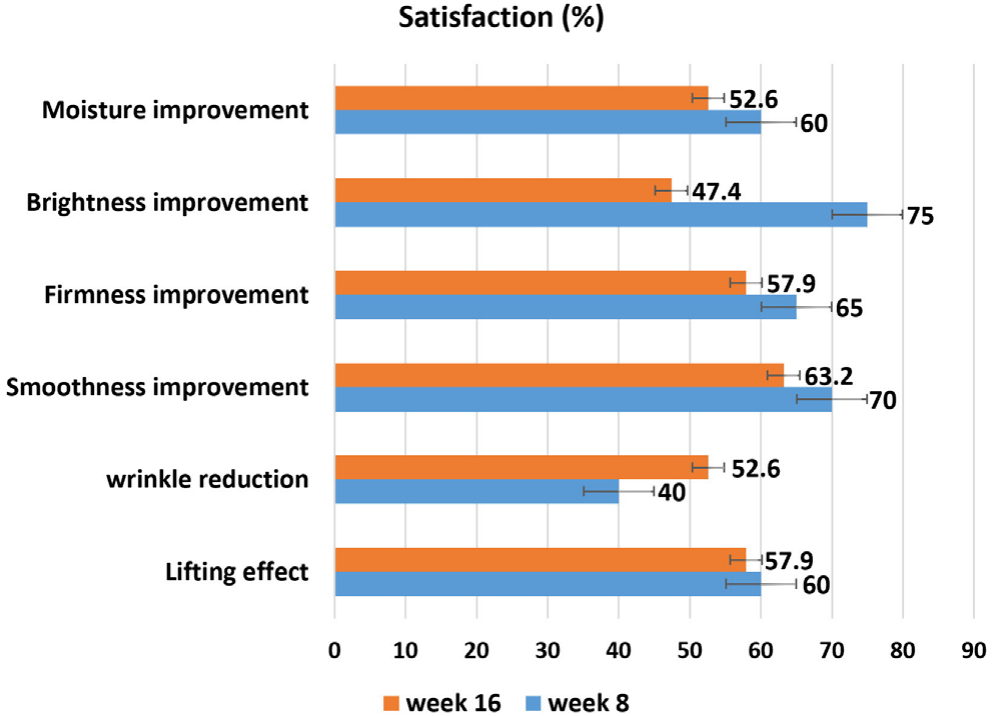
Participants' satisfaction based on the assessment of the skin conditions improvement. (Week 8 is first, and Week 16 is second follow‐up visit.)

The participants' reports of improvement were 60% for skin moisture, 75% for brightness, 65% for firmness, 70% for smoothness, 40% for wrinkle reduction, and 60% for lifting effect 4 weeks after the last treatment. The participants' reports of improvement were 52.6% for skin moisture, 47.4% for brightness, 57.9% for firmness, 63.2% for smoothness, 52.6% for wrinkle reduction, and 57.9% for lifting effect, 12 weeks after the last treatment (Figure 3).

The median overall satisfaction score was 7 (Min: 3, Max: 10) and 6 (Min: 1, Max: 10) at Weeks 4 and 12 after the last treatment, respectively.

**FIGURE 4:**
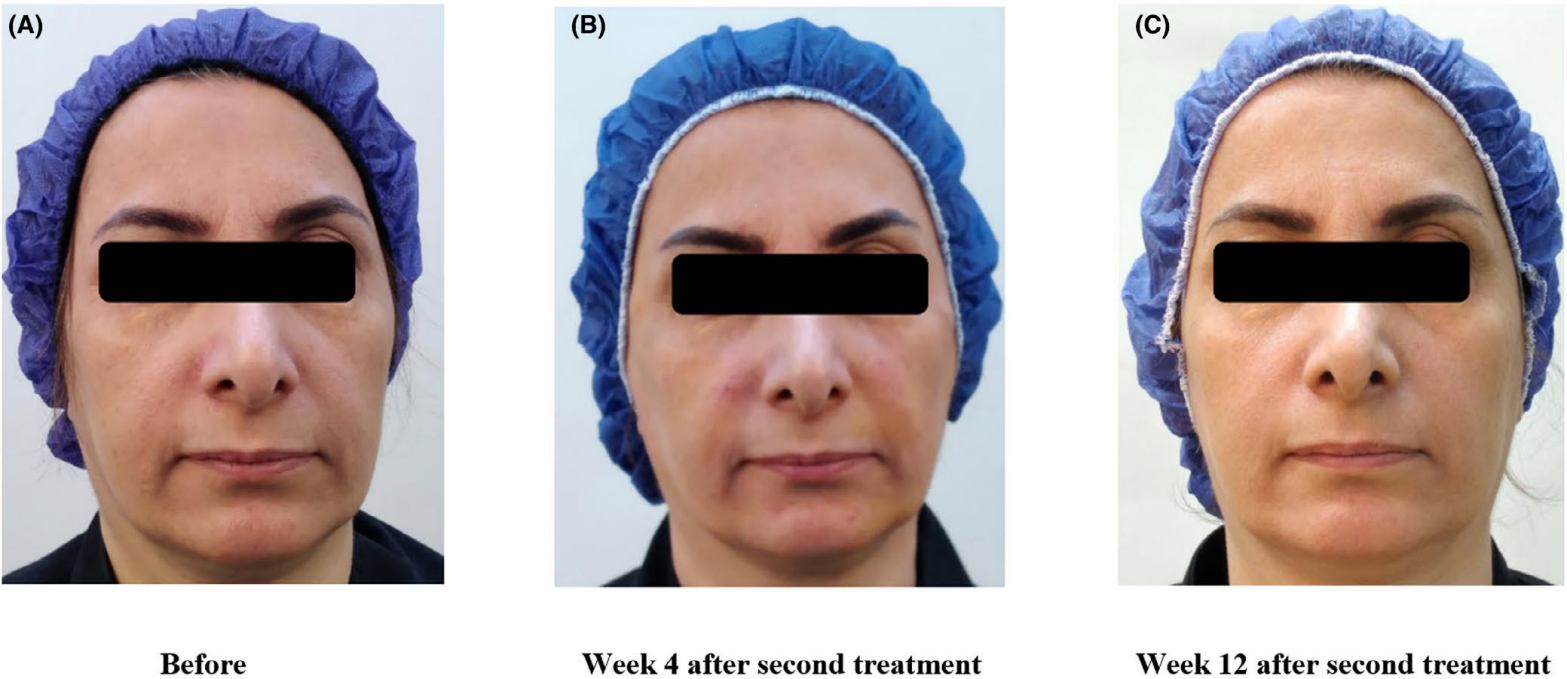
Representative images of a 56‐year‐old woman. (A: Before/B: Week 4 after second treatment/C: Week 12 after second treatment.)

The median GAIS score at both visits was 3 (Min: 2, Max: 4), which indicates an average improvement of 51%–75% in the participants. As seen in Table 2, all participants showed a 25% or greater improvement in skin aging signs according to an independent physician scoring, using the before‐and‐after photographs, at both visits. Representative images of one of the participants are shown in Figure 4.

**TABLE 2:** Assessment of the Global Aesthetic Improvement Scale.

GAIS score	Week 4 after second treatment		Week 12 after second treatment	
	*N*	%	*N*	%
1:< 25% improvement	0	0	0	0
2: 25%–50% improvement	5	25.0	3	15.8
3: 51%–75% improvement	11	55.0	11	57.9
4: More than 75% improvement	4	20.0	5	26.3
Total	20	100.0	19	100.0

Adverse events included nodule formation in the tragus region after the first injection in a 52‐year‐old woman, which resolved after 1 week. No other adverse events, including skin irritation reactions, such as redness, itching, bruising, swelling, and scaling, were observed or reported during the study course.

The median pain of the patients during both injections was 2 out of 10, based on the VAS score. The maximum pain score was reported as 9 in the first injection and 8 in the second injection.

## 4 Discussion

According to the results of this study, two face bioaesthetic points treatment with the test high and low molecular weight hyaluronic acid hybrid complex resulted in a significant improvement in total elastic and plastic deformation of skin or its firmness. Also, TEWL, dermis thickness, and density improved. In addition, we demonstrated safety, tolerance, and volunteer satisfaction with the product.

This novel method counteracts the physiological reduction of HA, improving hydration, skin tone, and elasticity. A clinical study conducted in 2015 on female users of a product with the same formulation showed that two treatment sessions significantly improved the S.C hydration, elasticity, and TEWL of the facial skin [14]. Also, another study conducted by Sparavigna et al. on 64 women aged 38–60 years showed biophysical improvements in the skin up to 3 months after the second injection [13].

In the current study, R0 (skin firmness) as one of the most important parameters of skin elasticity (primary outcome) improved significantly at both follow‐up visits. R2 (gross elasticity) and R5 (net elasticity) showed non‐significant increases at the last follow‐up visit; a larger sample size might make these changes statistically significant. Skin friction improved too, but non‐significantly. TEWL decreased significantly in the first follow‐up visit, and S.C hydration increased non‐significantly in both visits; we believe that a greater sample size would make this increase significant because TEWL reduced significantly, and these two parameters correlate reversely. The integrity improvement of the barrier function is typically shown by TEWL decrease, which is related to S.C hydration increase [15]. Evaluation of S.C hydration detects the efficacy of an anti‐aging product in terms of enhanced water content in the upper portion of the S.C under constant moisture and temperature conditions [16, 17].

Two other studies that used hybrid cooperative HA complexes technology showed amelioration in skin texture, hydration, smoothing, elasticity, brightness, and tone, as well as improvement in wrinkles and fine lines [18, 19].

Due to NAHYCO Hybrid Technology as a specific and patented treatment, the H‐HA and L‐HA chains in the product interact with each other and provide unique rheological characteristics, including appropriate HA concentration, low viscosity, and good tissue distribution for multilevel tissue remodeling, which is due to the administration of higher concentrations of HA without increasing the viscosity [20]. Most of the HA‐based products used in dermatology are based on chemical cross‐linking [21], which alters the natural structure of HA, despite considerably improving elasticity, stability, and firmness. NAHYCO Hybrid Technology is an innovative patented thermal production process to stabilize the HA without the use of cross‐linking agents [22].

The changes in thickness of the dermis layer at 12th week and its ecodensity at 4th week after the last treatment showed a statistically significant increase. This increase in the first weeks is due to the entry of the gel into the dermis space, but in the last weeks, it can indicate the synthesis of skin fibers, including collagen and elastin [23]. High‐frequency ultrasound is an important assessment device for the efficacy of anti‐aging products, and different HA fillers show various diffusion patterns in skin tissues by it [24]. For example, Laurino's post‐treatment echographic evaluation showed a widening of the deep dermal thickness after two hybrid cooperative HA complexes injections [14].

An average improvement of 51%–75% was reported by independent physicians, and all participants showed at least a 25% improvement in skin aging signs, which indicates appropriate product efficacy. The good efficacy of hybrid cooperative HA complexes for facial rejuvenation is also shown by some other in vivo studies [13, 14, 18, 19, 25, 26].

More than 60% of participants reported improvement in most skin conditions 1 month after the last treatment session. Only wrinkle reduction was reported by less than half of the study participants. A smaller percentage of participants in the Sparavigna study reported these improvements [13]. The median overall satisfaction scores were 7 (Week 4 after the second treatment) and 6 (Week 12 after the second treatment). All of these show appropriate subject satisfaction.

The median pain VAS score was 2, which shows that the treatment process was completely tolerable. The HA, at both low and high molecular weights used in the test product, is obtained through a bio‐fermentation process without chemical modification, resulting in excellent tolerability [27, 28]. Patient comfort and the absence of undesirable effects are important in aesthetic procedures [29].

Some studies have shown both patients' and physicians' high satisfaction levels of hybrid cooperative HA complexes [13, 14, 18, 19, 25, 26]. The current research confirmed the excellent outcome, without major and minor side effects. The only complication observed in the study was nodule formation in the tragus region in one of the participants after the first injection, which resolved after 1 week, showing a good safety profile. In a 2020 study on a similar filler injection, reported side effects included mild and temporary redness, itching, bruising, swelling, and scaling in 2.3% of the injections which all of which recovered within a week [26]. Even such minor complications were not observed in our study.

In terms of the strengths of the study, we performed a comprehensive safety and efficacy evaluation for a HA‐based formulation with new production technology in Iranian people with Fitzpatrick skin types III and IV. The interesting finding in our study was the significant increase in skin firmness and thickness, 3 months after the last treatment session. These findings could indicate the long‐lasting and continuous efficacy of hybrid cooperative HA complex injections. The limitation of the study was the lack of a control group; the participants were considered as their controls. A small sample size and no deeper skin hydration assessments with other objective tools could be considered as other limitations. Moreover, a longer follow‐up period is essential to determine whether the observed improvements are sustained over time. Totally larger, controlled studies with a longer follow‐up period are highly suggested for the future.

## 5 Conclusion

Based on the results, two injections of the test product (a stabilized HA injection gel) are a safe and well‐tolerated treatment to improve skin biophysical characteristics, especially its firmness, thickness, and density. This new HA‐based formulation is an appropriate clinical treatment option to restore vitality and turgidity of the skin and to induce skin amelioration. A larger controlled study evaluation can be designed to demonstrate the best treatment protocol for achieving the most favorable clinical outcomes.

## Author Contributions

Aniseh Samadi and Alireza Firooz performed the research. Azin Ayatollahi, Alireza Firooz, and Taraneh Yazdanparast designed the research study. Hamidreza Kafi, Taraneh Yazdanparast, and Araz Sabzvari contributed essential reagents or tools. Aniseh Samadi analyzed the data. Taraneh Yazdanparast and Alireza Firooz wrote the paper.

## Conflicts of Interest

Araz Sabzvari is a member of CinnaGen medical biotechnology research center, which collaborates with universities and researchers all over the world with regards to research and development of medications and health issues. Hamidreza Kafi is the head of the medical department of Orchid Pharmed Company which is in collaboration with Espad Pharmed Daru Company with respect to conducting clinical trials. Other authors declare no conflicts of interest.

## Data Availability

The data that support the findings of this study are available on request from the corresponding author. The data are not publicly available due to privacy or ethical restrictions.
